# HUWE1 and TRIP12 Collaborate in Degradation of Ubiquitin-Fusion Proteins and Misframed Ubiquitin

**DOI:** 10.1371/journal.pone.0050548

**Published:** 2012-11-27

**Authors:** Esben G. Poulsen, Cornelia Steinhauer, Michael Lees, Anne-Marie Lauridsen, Lars Ellgaard, Rasmus Hartmann-Petersen

**Affiliations:** 1 Department of Biology, University of Copenhagen, Copenhagen, Denmark; 2 Biotech Research and Innovation Centre, University of Copenhagen, Copenhagen, Denmark; University of Toronto, Canada

## Abstract

In eukaryotic cells an uncleavable ubiquitin moiety conjugated to the N-terminus of a protein signals the degradation of the fusion protein via the proteasome-dependent ubiquitin fusion degradation (UFD) pathway. In yeast the molecular mechanism of the UFD pathway has been well characterized. Recently the human E3 ubiquitin-protein ligase TRIP12 was connected with the UFD pathway, but little is otherwise known about this system in mammalian cells. In the present work, we utilized high-throughput imaging on cells transfected with a targeted siRNA library to identify components involved in degradation of the UFD substrate Ub^G76V^-YFP. The most significant hits from the screen were the E3 ubiquitin-protein ligase HUWE1, as well as PSMD7 and PSMD14 that encode proteasome subunits. Accordingly, knock down of HUWE1 led to an increase in the steady state level and a retarded degradation of the UFD substrate. Knock down of HUWE1 also led to a stabilization of the physiological UFD substrate UBB^+1^. Precipitation experiments revealed that HUWE1 is associated with both the Ub^G76V^-YFP substrate and the 26S proteasome, indicating that it functions late in the UFD pathway. Double knock down of HUWE1 and TRIP12 resulted in an additive stabilization of the substrate, suggesting that HUWE1 and TRIP12 function in parallel during UFD. However, even when both HUWE1 and TRIP12 are downregulated, ubiquitylation of the UFD substrate was still apparent, revealing functional redundancy between HUWE1, TRIP12 and yet other ubiquitin-protein ligases.

## Introduction

The ubiquitin-proteasome system (UPS) is responsible for the majority of intracellular protein degradation, and also plays an important regulatory role in many cellular processes, including metabolism, cell division, DNA repair, antigen presentation, signal transduction, and development [1].

With few exceptions, proteins must be conjugated to a chain of ubiquitin before they become substrates for the 26S proteasome. The conjugation reaction relies on a cascade of three enzymes, termed E1, E2, and E3 that conjugate the small protein ubiquitin to specific target proteins [1]. In this enzyme cascade the ubiquitin-activating enzyme (E1) first activates ubiquitin in an ATP-dependent reaction by forming a thioester bond with the C-terminal glycine residue (G76) in ubiquitin. Ubiquitin is then transferred to the ubiquitin-conjugating enzyme (E2) before a ubiquitin-protein ligase (E3) transfers ubiquitin to a lysine residue in the target protein. Ubiquitin can also be transferred to a lysine residue in ubiquitin already attached to the target protein, thus forming a chain of ubiquitin moieties. In some cases the ubiquitin chain on the target protein may be elongated by an E4 ubiquitin-chain elongation factor [2].

Depending on their enzymatic domains and reaction mechanism, E3s are generally classified into two major families: RING (really interesting new gene) domain E3s and HECT (homologous to E6AP C-terminus) domain E3s. The HECT domain E3s normally contain a diverse N-terminal region and a conserved C-terminal HECT domain. The HECT domain contains a reactive cysteine residue that forms a transient thioester bond to the ubiquitin C-terminal carboxyl group before transfer of the ubiquitin moiety to the target protein [3]. RING domains, on the other hand, do not form covalently-linked intermediates with ubiquitin during the reaction.

The process of ubiquitylation is reversible, and the human genome encodes around 100 different deubiquitylating enzymes (DUBs), which can all potentially trim or release ubiquitin chains conjugated to target proteins [4].

The proteins that have been marked with polyubiquitin are targeted to the 26S proteasome, a large and abundant proteolytic particle found in the nucleus and cytosol of all eukaryotic cells [5]. At the 26S proteasome, the ubiquitin chains can be further elongated or released, while the substrate is degraded into shorter peptides that can be displayed on the cell surface for immuno surveillance or further degraded to free amino acids by various amino peptidases [6].

Although it was first described in yeast [7] [2], the ubiquitin fusion degradation (UFD) pathway is conserved in all eukaryotic cells [8]. In this proteolytic system, an uncleavable ubiquitin moiety, fused to the N-terminus of a protein, signals rapid degradation of the fusion protein. Elegant studies have defined the key components of this pathway in the budding yeast, *Saccharomyces cerevisiae* [7] [2]. Ubiquitylation is catalyzed by the HECT domain E3, called Ufd4 [9], while ubiquitin chain elongation is performed by the E4, Ufd2 [2]. In addition, the UFD pathway requires the AAA (ATPase associated with various cellular activities) ATPase, called Cdc48 in yeast and p97 or VCP in mammals, and the p97/Cdc48 co-factors Ufd1 and Ufd3 [7] [10]. So far most studies on the UFD system have utilized artificial model substrates. However, more recently the human orthologue of budding yeast Ufd4, called TRIP12, was shown to participate in the UFD pathway and mediate degradation of UBB^+1^, a physiological UFD substrate [11]. UBB^+1^ is a frame shift mutant ubiquitin derived from a dinucleotide deletion in the mRNA of the *ubiquitin B* gene, leading to an uncleavable N-terminal ubiquitin moiety linked to a short C-terminal extension [12]. In patients with neurodegenerative disorders, including Alzheimer’s diseases and various polyglutaminopathies, UBB^+1^ accumulates in brain tissue [13]. Studies have shown that high levels of UBB^+1^ inhibits the UPS and leads to cell cycle arrest [14]. Thus, high concentrations of UBB^+1^ will most likely contribute to an environment that favors accumulation of misfolded proteins and in this manner further aggravate the disease. However, at lower levels UBB^+1^ is efficiently cleared via the UFD pathway [14]–[15] [11].

To determine if other cellular E3s collaborate with TRIP12 in the degradation of UFD substrates, we utilized a targeted siRNA screen to identify novel components involved in degradation of the ubiquitin fusion protein Ub^G76V^-YFP. The most significant hits from the screen included the E3 ubiquitin-protein ligase HUWE1 and 26S proteasome subunits. Accordingly, knock down of HUWE1 led to a retarded degradation of the Ub^G76V^-YFP and UBB^+1^ substrates. Since we could co-precipitate HUWE1 with the substrate and the 26S proteasome, we propose that HUWE1 directly ubiquitylates UFD substrates at a late step during the degradation pathway.

**Figure 1:**
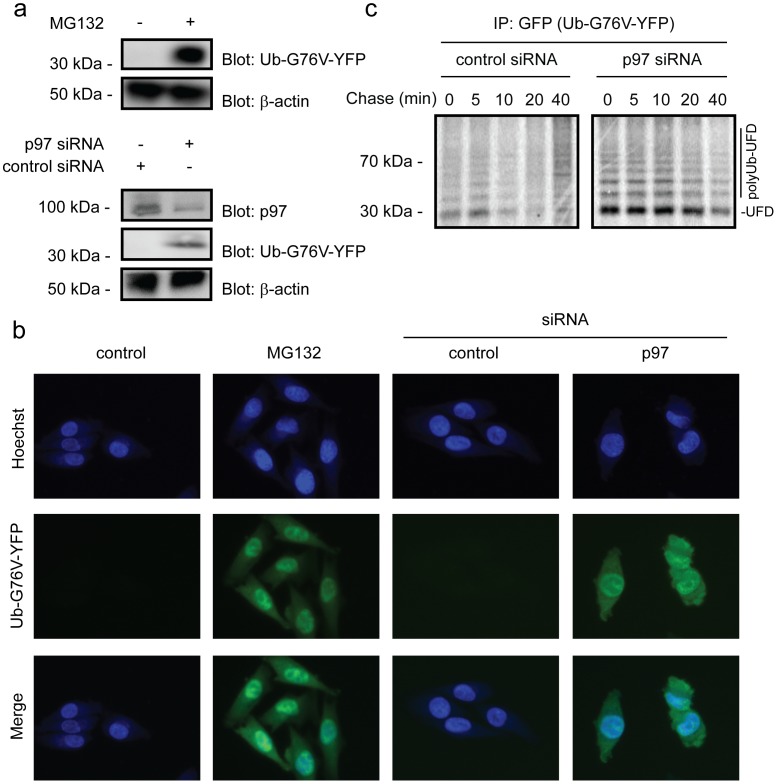
The proteasome and p97 are required for Ub^G76V^-YFP degradation. (a) The steady state level of the Ub^G76V^-YFP substrate was determined by blotting extracts from cells treated with the proteasome inhibitor MG132 and siRNA to p97. Actin served as a loading control. (b) Fluorescence microscopy of Ub^G76V^-YFP (green) in cells treated with the proteasome inhibitor MG132 or siRNA to p97. Hoechst staining (blue) was used to mark the nuclei. (c) Pulse-chase analyses of Ub^G76V^-YFP degradation in cells transfected with control siRNA or siRNA specific for p97.

## Materials and Methods

### Buffers, Plasmids and Antibodies

Buffer A, 25 mM Tris/HCl pH 7.5, 2 mM MgCl_2_, 2 mM ATP, 50 mM NaCl, 1 mM DTT, 10% (v/v) glycerol, 0.1% (v/v) Triton X-100.

Plasmids for expression of flag-tagged UBB^+1^ were generously supplied by Prof. Yoon, Yonsei University, Korea.

The antibodies to p97 have been described previously [16]. Antibodies to human proteasomes were purchased from Enzo. The anti-GFP, anti-flag and anti-β-actin antibodies were purchased from Sigma. Antibodies to HUWE1 have been described previously [17] and were kindly supplied by Prof. Wing, McGill University, Canada. All secondary antibodies were purchased from Dako.

### Cell Culture

MelJuso cells, stably transfected to express ubiquitin-G76V-YFP [18], were maintained in Dulbecco’s modified eagle’s media (DMEM) containing 10% fetal bovine serum (Biosera), and supplemented with glutamine, penicillin/streptomycin and 0.25 mg/ml geneticin at 37°C in a humidified atmosphere containing 7.5% CO_2_.

### Electrophoresis and Blotting

Proteins were separated on 7 cm × 8 cm 12.5% or 8% acrylamide gels. Proteins were transferred to BA83 (Schleicher & Schuell) nitrocellulose membranes and probed with antibodies as indicated.

### Transfection

Interfering RNAs specific for human HUWE1, TRIP12 and p97 were purchased as either siRNA #1 ON-TARGETplus SMARTpool from Thermo scientific (HUWE1: J-00718507, J-00718508, J-00718509, J-00718510. TRIP12: J-00718206, J-00718207, J-00718208, J-00718209. VCP/p97: D-00872705, D-00872706, D-00872707, D-00872708. Non-targeting pool: D-00181010) or siRNA #2 FlexiTube siRNA (SI04336178) from Qiagen. The siCONTROL siRNA#1 (Dharmacon) was used as an unspecific control. Trypsinized cells were incubated for 48 hours with a solution containing 50 nM siRNA, 0.001% lipofectamin RNAi MAX in 40% OptiMEM and 60% DMEM supplemented with 10% fetal bovine serum.

**Figure 2:**
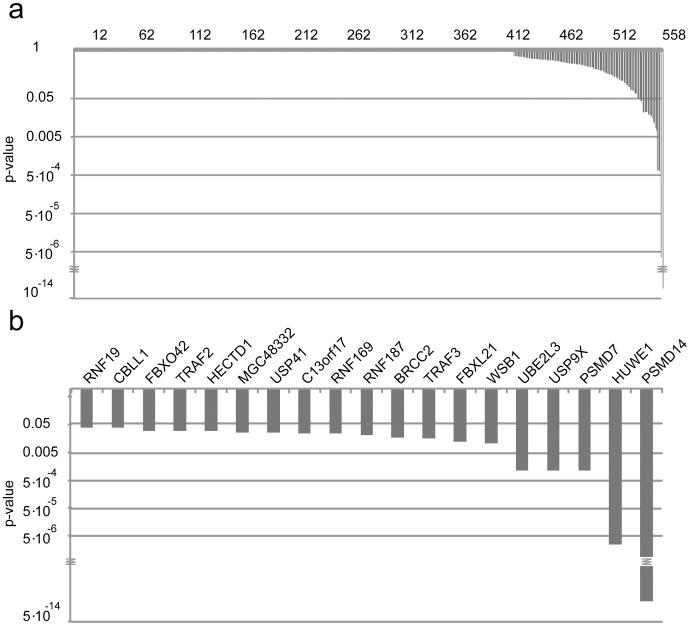
Results from the siRNA screens. (a) The 558 different genes assayed in the screen ranked according to the p-values assigned to each gene using the RSA analysis and the Fisher's probability test. Each gene is assigned a single p-value based on the ranking distribution of all three siRNAs in three individual screening experiments. (b) The genes scoring a combined p-value below 5% are ranked.

**Table 1:** Significantly scoring genes.

Gene	Protein	Fisher’s combined p-values
*PSMD14*	proteasome 26S subunit, non-ATPase 14	2.1E-14
*HUWE1*	HECT, UBA and WWE domain containing 1	2.7E-06
*PSMD7*	proteasome 26S subunit, non-ATPase 7	0.0013
*USP9X*	ubiquitin specific protease 9, X-linked	0.0014
*UBE2L3*	ubiquitin-conjugating enzyme, E2L3	0.0014
*WSB1*	WD repeat and SOCS box-containing 1	0.012
*FBXL21*	F-box and leucine-rich repeat protein 21	0.014
*TRAF3*	TNF receptor-associated factor 3	0.018
*BRCC2*	BRCC2	0.019
*RNF187*	ring finger protein 187	0.024
*RNF169*	ring finger protein 169	0.026
*C13orf7*	chromosome 13 open reading frame 7	0.028
*USP41*	ubiquitin specific protease 41	0.028
*MGC48332*	membrane-associated ring finger (C3HC4) 3	0.029
*HECTD1*	HECT domain containing 1	0.032
*TRAF2*	TNF receptor-associated factor 2	0.032
*FBXO42*	F-box protein 42	0.033
*CBLL1*	Cas-Br-M	0.042
*RNF19*	ring finger protein 19	0.043

### Co-precipitations

For precipitation experiments Mel-Juso cells, stably transfected to express Ub^G76V^-YFP, were treated for 6 hours with 25 µM MG132 and then lyzed by sonication in 4 volumes of buffer A. Cleared extracts were prepared by centrifugation (12000 g, 30 min). The cleared lysates were incubated at 4°C for 4 hours with gentle agitation with 0.25 µL anti-GFP (Sigma) or 1 µL anti-MCP20 (Enzo) and 20 µL Protein G Sepharose beads (GE Healthcare). As a negative control antibodies to α2-macroglobulin were used. The beads were then washed in 3 × 15 mL of buffer A before elution in SDS sample buffer. The eluted material was analyzed by SDS-PAGE and immunoblotting.

### Protein Degradation Experiments

The stability of the Ub^G76V^-YFP model substrate was followed by pulse-chase analysis on stably transfected cells expressing the substrate, as described previously [19]. The degradation of UBB^+1^ was followed by cycloheximide decay assays as described [11].

### Reverse Transcription PCR

The knock down efficiency of TRIP12 was determined by reverse transcription PCR. For this purpose mRNA was isolated using TRIzol (Invitrogen) and Turbo DNA-free (Ambion). The RNA was reverse transcribed using Transcriptor first strand cDNA synthesis (Roche Applied Science), purified using GFXPCRDNA (GE Healthcare), and used for PCR. GAPDH was used as a negative control. The primers specific for GAPDH were: forward, AGCCTCCCGCTTCGCTCTCT, reverse, TGACCTTGGCCAGGGGTGCT. The primers specific for TRIP12 were: forward, CCGGGGCCCAACCACAAGAC, reverse, TGGACGCTGAACGGGAACGC.

### siRNA Screening

The siRNA library used was a customized Human Silencer siRNA Library from Applied Biosystems. The library consists of three individual siRNAs specific for 558 different gene products predicted to play a role in the ubiquitin-proteasome system. A list of all targets in the library can be found under supplementary materials (Table S1).

The automated screen was performed using the MicrolabSTAR liquid handling system (Hamilton Robotics). MelJuso cells were reverse transfected with the customized siRNA library including individual positive and negative controls. In brief, 4 µL siRNA was added to 2.5 µL OptiMEM (Invitrogen) into each well of a 384-well plate (Corning) (Fig. S1). To this, a 6.4 µL OptiMEM/0.1 µL RNAiMAX (Invitrogen) mix was added and left for 15 min, after which 27 µL of cells were added to give a total cell density of 2400 cells per well. The final concentration of siRNA was 50 nM. Cells were then incubated for 48 h or 72 h, followed by fixation (Gurr histological fixative, VWR) and Hoechst staining (Sigma-Aldrich). In addition, individual wells were treated with 25 µM MG132 4 hours prior to fixation. Plates were imaged on an IN Cell Analyzer 1000 (GE Healthcare) acquiring a minimum of 6 images per well using a 10× objective to count approximately 2000 cells per well.

**Figure 3:**
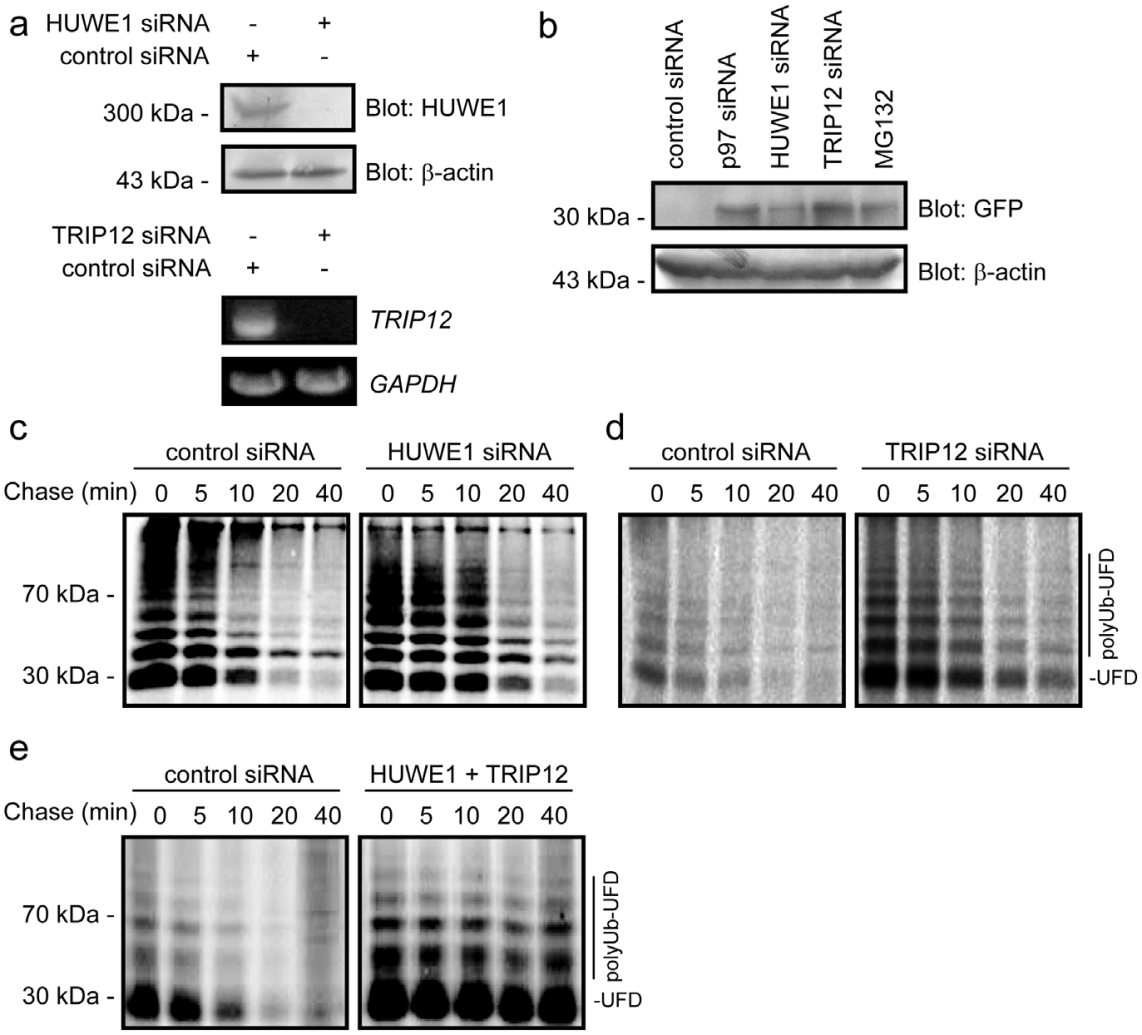
HUWE1 and TRIP12 are required for Ub^G76V^-YFP degradation. (a) Efficient knock down of HUWE1 was validated by Western blotting, while for TRIP12 knock down was validated by RT-PCR. Actin and GAPDH served as loading controls. (b) The steady state level of the Ub^G76V^-YFP substrate was determined in cultures by blotting with antibodies to GFP. Actin served as a loading control. (c) Pulse-chase analyses of Ub^G76V^-YFP degradation in cells transfected with control siRNA or siRNA specific for HUWE1. (d) Pulse-chase analyses of Ub^G76V^-YFP degradation in cells transfected with control siRNA or siRNA specific for TRIP12. (e) Pulse-chase analyses of Ub^G76V^-YFP degradation in cells transfected with control siRNA or siRNA specific for TRIP12 and HUWE1.

### Image Analysis and Statistics

Acquired images were analyzed using the IN Cell Analyzer Workstation 3.6 software (GE Healthcare) and percentage of ubiquitin^G76V^-YFP positive cells scored based on the overall nuclear and cellular intensity. This data was then normalized to the median sample score of each plate (excluding controls) and a gene-based hit list was generated using the statistical method “redundant siRNA activity” (RSA) analysis [20]. The RSA method first ranked individual siRNAs according to their normalized scores (excluding controls) and then calculated p-values for each gene based on the likelihood for this distribution of siRNA ranks to occur by chance. This calculation of p-values was based on the iterative hypergeometric distribution [20]. Subsequently, RSA-based p-values of the three individual screens were combined using Fisher's combined probability test. The entire statistical analysis was conducted using the statistical software ‘R’.

**Figure 4:**
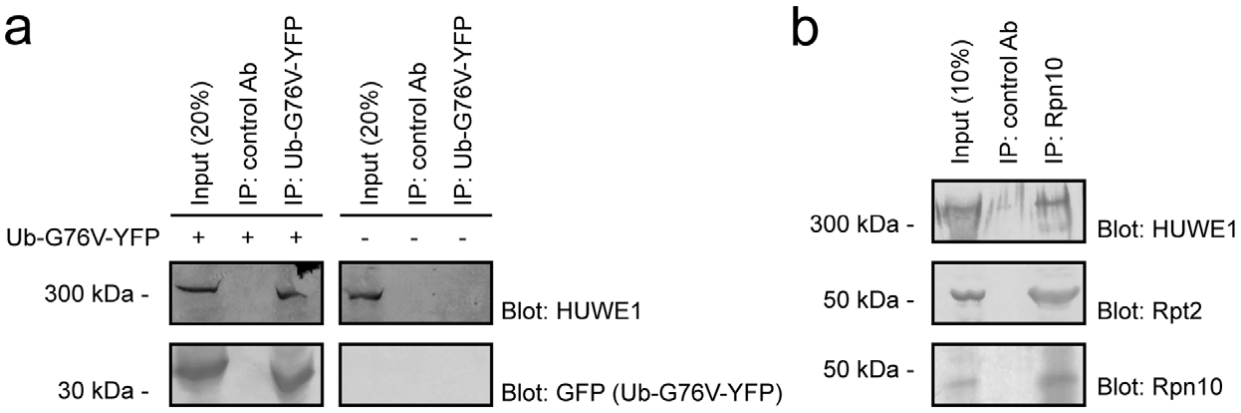
HUWE1 is associated with Ub^G76V^-YFP and the 26S proteasome. (a) Immuno precipitates (IP) of the Ub^G76V^-YFP substrate using either control antibodies or antibodies to GFP from cultures transfected to express Ub^G76V^-YFP (left panel) or as a control mock transfectants (right panel). The precipitates were analyzed by blotting with antibodies to HUWE1 and GFP. (b) 26S proteasomes were immunoprecipitated from MelJuSo cells using antibodies to Rpn10 and analyzed by blotting with antibodies to HUWE1 and the 26S proteasome subunits Rpn10 and Rpt2.

**Figure 5:**
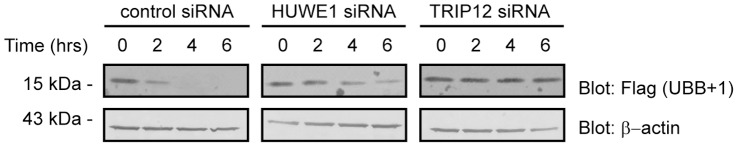
HUWE1 is required for UBB^+1^ degradation. The degradation of flag tagged UBB^+1^ was followed by blotting of cultures treated with cycloheximide (CHX) for the indicated times. Actin served as a loading control.

## Results

### p97 is Required for Degradation of Ub^G76V^-YFP

To study the UFD pathway, cells stably transfected to express the ubiquitin-G76V-YFP fusion protein (Ub^G76V^-YFP) were used [18]. In these cells, the steady-state level of the Ub^G76V^-YFP substrate was very low. By Western blotting using antibodies to GFP, we were unable to detect the Ub^G76V^-YFP protein in unperturbed cells (Fig. 1a). However, upon addition of the proteasome inhibitor MG132, a clear signal at about 35 kDa, corresponding to ubiquitin (∼8 kDa) and YFP (∼25 kDa), was obtained (Fig. 1a). This suggests that the low cellular level of the Ub^G76V^-YFP protein was due to rapid proteasomal degradation. By fluorescence microscopy, the Ub^G76V^-YFP protein was also undetectable in unperturbed cells, but again the level increased dramatically upon treatment with MG132 (Fig. 1b). In accordance with previous results [18], the fluorescent signal of Ub^G76V^-YFP appeared somewhat stronger in the nucleus than in the cytosol (Fig. 1b).

Since the AAA-type ATPase p97 is critical for degradation of many proteasome substrates, we also tested if knock down of p97 led to an increased level of the Ub^G76V^-YFP substrate protein. Indeed, we observed a robust increase in the Ub^G76V^-YFP level upon p97 knock down, both by Western blotting and by fluorescence microscopy (Fig. 1a and 1b). To determine the degradation kinetics of the UFD substrate, we performed pulse-chase analyses on cells transfected with siRNA to p97. In cells transfected with control siRNA we found that Ub^G76V^-YFP was rapidly degraded (Fig. 1c). As expected, p97 knock down strongly inhibited degradation (Fig. 1c), showing that p97 is critical for degradation of Ub^G76V^-YFP.

### Identification of Novel UFD Pathway Components by siRNA Screening

Since knock down of p97 led to a strong increase in the Ub^G76V^-YFP level, it was ideally suited as a positive control for a targeted siRNA screen. A customized siRNA library consisting of three individual siRNAs to each of 558 different gene products, predicted to play a role in the ubiquitin system (Table S1), was therefore reverse transfected into the reporter cell line. An siRNA targeting p97 and MG132 treatment were included as positive controls, whilst scrambled siRNA served as a negative control. After 48 or 72 hours the cells were fixed and the percentage of cells showing a YFP signal was monitored for each transfection by automated fluorescence microscopy. By subsequent image analysis, the percentage of cells displaying YFP signal was determined and plate-wise normalized. We observed no significant differences between the 48 and 72 hour transfection periods (Fig. S2). To minimize the risk of false scoring based on off-target effects of individual siRNAs, we computed a statistical score that modeled the probability of a gene ‘hit’ based on the collective activities of all three siRNAs per gene using the statistical method “redundant siRNA activity” (RSA) analysis [20]. Thus, RSA analysis scored genes based on a combined p-value of all siRNAs per gene. P-values of the three individual screens were then further combined to a common p-value based on Fisher’s probability test (Fig. 2a and Table S1). In total 19 genes, corresponding to about 3% of the library, scored significantly (Table 1). In addition to the p97 transfection control (Fig. S2), proteasome subunits PSMD7 and PSMD14 scored highly significant (Fig. 2b), suggesting that the screening approach worked. Another significant hit was the E3 ubiquitin-protein ligase HUWE1 (Fig. 2b).

### Slowed Ub^G76V^-YFP Degradation upon Knock Down of HUWE1

If HUWE1 identified from the siRNA screening indeed partakes in the degradation of the UFD substrate, one would expect that the degradation kinetics of the Ub^G76V^-YFP protein are slowed when HUWE1 is knocked down. To test this and thus verify the result of the high-throughput cellular imaging, pulse-chase experiments were performed.

Upon knock down of HUWE1, we observed a clear reduction in the cellular level of the HUWE1 protein (Fig. 3a). Since we were unable to obtain functional antibodies to TRIP12, successful depletion of this component was analyzed by RT-PCR (Fig. 3a). When expression of HUWE1, or as controls TRIP12 and p97, were knocked-down the steady-state level of the Ub^G76V^-YFP protein was increased (Fig. 3b). Accordingly, we observed that Ub^G76V^-YFP degradation upon knock down of HUWE1 was slowed (Fig. 3c). HUWE1, like TRIP12, is a HECT-type E3 ubiquitin-ligase. However, when HUWE1 expression was knocked down, ubiquitylation of the UFD substrate was still apparent (Fig. 3c), suggesting that other cellular E3s also target the substrate. We therefore decided to compare the effect of HUWE1 with that of TRIP12.

Knock down of TRIP12 also led to a stabilization of the Ub^G76V^-YFP substrate (Fig. 3d). Again, the ubiquitylation pattern of the UFD substrate did not appear to be affected. In double knock down cells, lacking both HUWE1 and TRIP12, the stability of the substrate was further increased (Fig. 3e), suggesting that HUWE1 and TRIP12 function in parallel. However, since ubiquitylation of the UFD substrate was still apparent (Fig. 3e), the function of HUWE1 and TRIP12 must overlap with yet other E3s.

### HUWE1 Associates with both the UFD Substrate and the 26S Proteasome

If HUWE1 directly regulates the degradation of Ub^G76V^-YFP, the two components should at least transiently form an enzyme-substrate complex. To test this prediction, we performed immuno-precipitation experiments with the Ub^G76V^-YFP substrate from cells treated with MG132. The precipitates were resolved by SDS-PAGE and analyzed by Western blotting. Probing the blots with antibodies to GFP revealed that the Ub^G76V^-YFP substrate was precipitated, but not present in the negative controls (Fig. 4a). During the course of these experiments, we also tested if 26S proteasomes associate with HUWE1. When immuno-precipitating the 26S proteasome, we observed that HUWE1 specifically co-precipitated (Fig. 4b).

### HUWE1 Stimulates Degradation of UBB^+1^

So far, the ubiquitin mutant UBB^+1^ constitutes the only physiologically relevant substrate of the UFD pathway [11]. In order to determine if HUWE1 also plays a role in UBB^+1^ degradation, the turnover of flag-tagged UBB^+1^ was followed in cultures treated with the translation inhibitor cycloheximide (CHX). UBB^+1^ was rapidly degraded in cells transfected with control siRNA and stabilized upon knock down of HUWE1 and TRIP12 (Fig. 5), revealing that HUWE1 also partakes in the degradation of UBB^+1^.

## Discussion

Bioinformatic analyses have shown that a significant fraction of the eukaryotic genome is dedicated to the UPS, in some species occupying as much as 5% of the genome [21]. This large number of genes of the UPS is mainly attributed to the many different E3 ubiquitin-protein ligases [21] that are the major factors in determining the specificity in conjugation of ubiquitin to target proteins. Since the human genome is predicted to encode more than 600 distinct E3s, the substrate specificity of the system should at least be of a similar diversity. A major challenge is to link the E3s with their respective substrate classes. This is not a trivial task, which is further complicated by a significant amount of cross talk between the degradation pathways. Thus, several proteins are ubiquitylated by different E3s. For instance, p53 is ubiquitylated by multiple E3s, including Mdm2, Hrd1/Synoviolin and CHIP [22].

In the present study, we utilized high-throughput cellular imaging on cells transfected with a targeted siRNA library to screen for components involved in degradation of ubiquitin fusion proteins. This is a powerful tool for studying gene function and mapping biochemical pathways, which has recently been successfully applied to study various aspects of ubiquitin-dependent protein degradation, including identification of protein quality control factors [23].

The success of the screen was readily apparent since two proteasome subunits PSMD7 and PSMD14 appeared as highly significant hits. Our targeted siRNA library did not include any other proteasome subunits, but presumably knockdown of most proteasome subunits would interfere with the UFD pathway. The second best scoring hit was the HECT-type E3 ubiquitin-protein ligase HUWE1 (also known as ARF-BP1, Mule, HECTH9, E3Histone, LASU1 and UREB1), recently connected with X-linked mental retardation [24]. Since we could co-precipitate HUWE1 with the Ub^G76V^-YFP model substrate, we suggest that HUWE1 directly ubiquitylates this protein for subsequent degradation. Several HUWE1 targets, including signaling proteins [25]–[26] and core histones [27], have previously been identified, but this is the first demonstration of HUWE1 functioning in the UFD pathway.

The E2 called UBE2L3 was another significant hit in our screen. This is noteworthy since UBE2L3 (also known as UbcH7) is an E2 enzyme which displays specificity for HECT type E3s and has been reported to associate with HUWE1 [25] [28]–[29]. Studies have shown that UBE2L3 functions as the cognate E2 for HUWE1 catalyzed ubiquitylation of the anti-apoptotic Mcl-1 protein [25]. It is therefore possible that UBE2L3 functions as E2 enzyme in the UFD pathway.

In pulse-chase analyses, knock down of HUWE1 and the human orthologue of Ufd4, TRIP12, led to a comparable stabilization of the UFD substrate. In addition to the C-terminal HECT domains, both HUWE1 and TRIP12 also contain WWE domains [3], but do otherwise not share sequence similarity. However, since studies have shown that the TRIP12 HECT domain alone can mediate degradation of UFD substrates [11] it is possible that other HECT-type E3s will function in a similar fashion. TRIP12 was not included in our siRNA library, but indeed we note that another HECT domain E3, HECTD1 (Fig. 2b), was a significant hit in our screen. However, various RING-type E3s also scored significantly, while several other HECT domain E3s, included in the siRNA library, failed to score.

Since the UFD pathway is phylogenetically conserved in eukaryotes [8], it is surprising that UBB^+1^ so far is the only known UFD substrate, which is physiologically relevant. However, HUWE1 has recently been shown to catalyze N-terminal (α-amino) ubiquitylation, thus forming linear ubiquitin fusion proteins [26]. It is therefore conceivable that HUWE1 and other E3 ligases in this manner generate natural UFD substrates.

Previous studies have shown that TRIP12 mediates ubiquitylation and degradation of UBB^+1^ [11], and our data show that this function is shared with HUWE1. However, in comparison with TRIP12 the effect of HUWE1 shown here appears more moderate. Previously, a proteomics study found that HUWE1 associates with 26S proteasomes from brain tissue [30]. We also found that HUWE1 co-precipitates with 26S proteasomes, suggesting it might function at a rather late step in the degradation pathway, where perhaps several degradation pathways converge. Given that HUWE1 contains several ubiquitin binding domains it is possible that it may display specificity for already ubiquitylated substrates and could therefore function in ubiquitin chain elongation similar to the E4 Ufd2 [2].

## Supporting Information

Figure S1 **siRNA Library Overview.** The figure depicts the layout of the siRNA library plates utilized.
(TIF)

Figure S2 **siRNA screen performance.** Overall screen performance based on the percentage of Ub^G76V^-YFP positive cells in non-silencing control (N), positive control p97 (P) and individual siRNAs from the screening library (S). Percentages were plate-wise normalized and arcsinh transformed for better clarity.
(TIF)

Table S1 **Statistical analyses of the siRNA screens.** P-values of the three individual screens combined to a common p-value based on Fisher’s probability test for all library targets.
(XLS)
