# Supplementary figures and images for: HUWE1 and TRIP12 Collaborate in Degradation of Ubiquitin-Fusion Proteins and Misframed Ubiquitin
Source: PLoS One. 2012 Nov 27;7(11):e50548. doi: 10.1371/journal.pone.0050548 (PMC3507821; doi:10.1371/journal.pone.0050548)

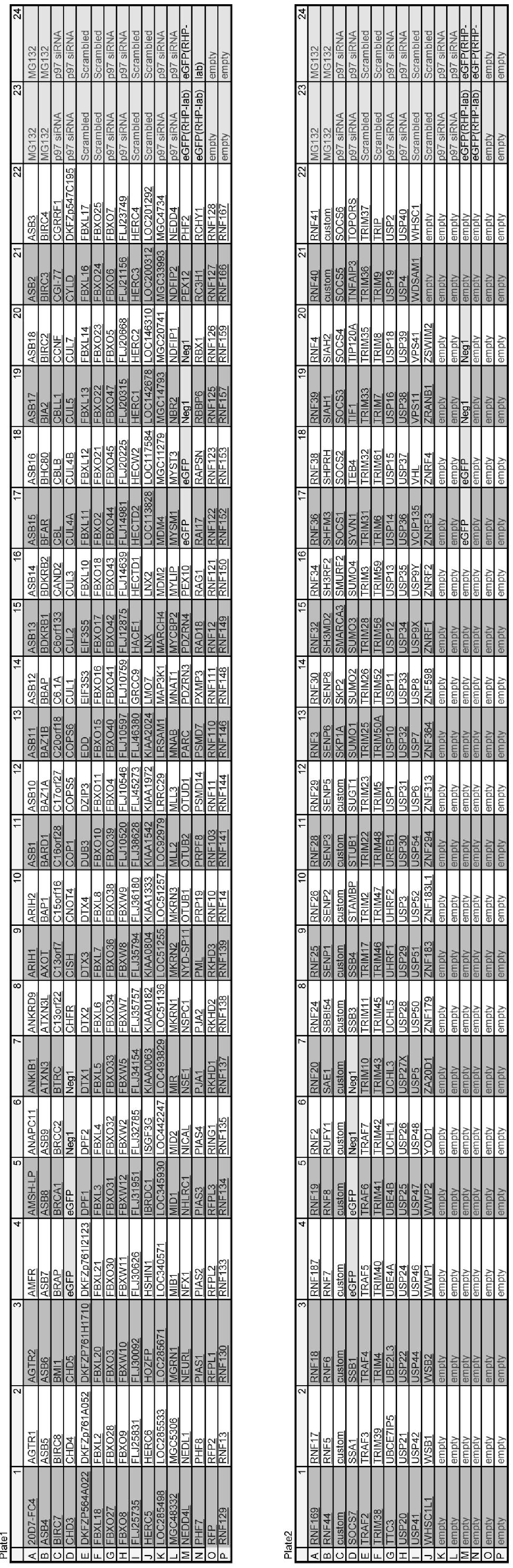

Supplement: Figure S1 — siRNA Library Overview. The figure depicts the layout of the siRNA library plates utilized. (TIF) [file pone.0050548.s001.tif]

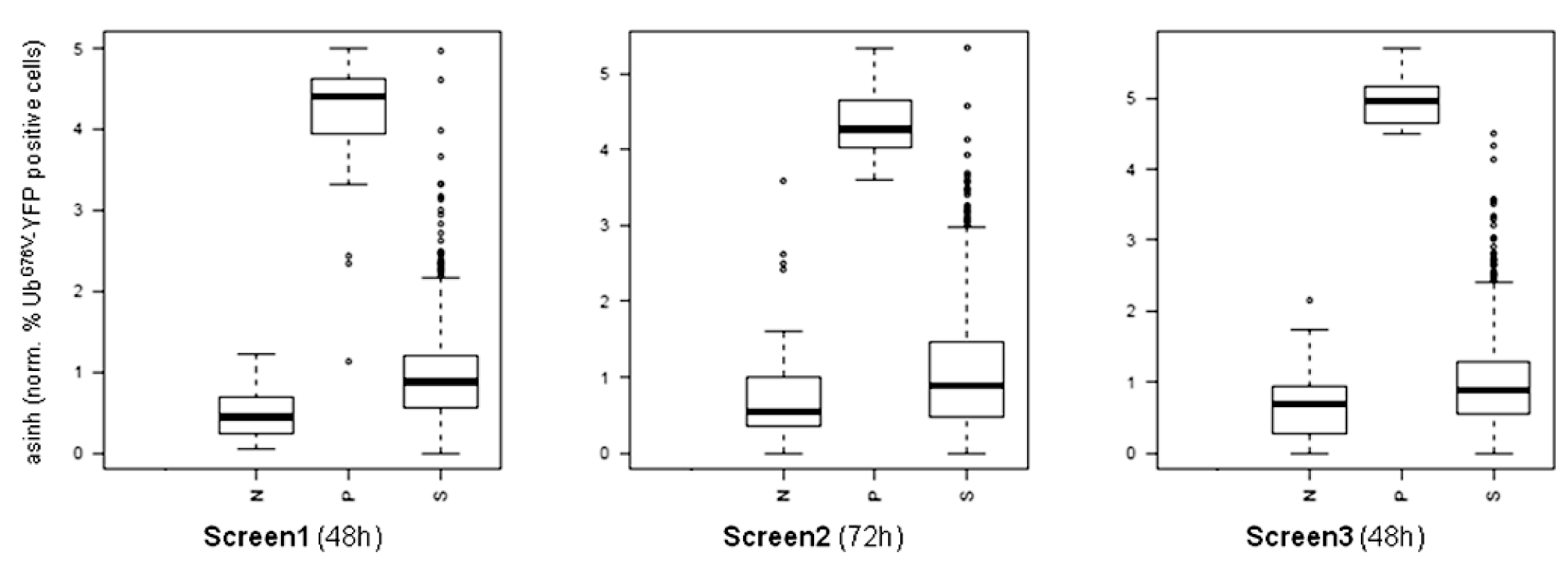

Supplement: Figure S2 — siRNA screen performance. Overall screen performance based on the percentage of UbG76V-YFP positive cells in non-silencing control (N), positive control p97 (P) and individual siRNAs from the screening library (S). Percentages were plate-wise normalized and arcsinh transformed for better clarity. (TIF) [file pone.0050548.s002.tif]
